# Effects of probiotics on neurocognitive outcomes in infants and young children: a meta-analysis

**DOI:** 10.3389/fpubh.2023.1323511

**Published:** 2023-12-05

**Authors:** Feng-Li Lin, Chia-Min Chen, Cheuk-Kwan Sun, Yu-Shian Cheng, Ruu-Fen Tzang, Hsien-Jane Chiu, Ming-Yu Wang, Ying-Chih Cheng, Kuo-Chuan Hung

**Affiliations:** ^1^Taoyuan Psychiatric Center, Ministry of Health and Welfare, Taoyuan, Taiwan; ^2^Department of Natural Biotechnology, Nanhua University, Chiayi, Taiwan; ^3^Department of Emergency Medicine, E-Da Dachang Hospital, I-Shou University, Kaohsiung, Taiwan; ^4^School of Medicine for International Students, College of Medicine, I-Shou University, Kaohsiung, Taiwan; ^5^Department of Psychiatry, Tsyr-Huey Mental Hospital, Kaohsiung Jen-Ai’s Home, Kaohsiung, Taiwan; ^6^Department of Psychiatry, Mackay Memorial Hospital, Taipei, Taiwan; ^7^Institute of Hospital and Health Care Administration, National Yang-Ming University, Taipei, Taiwan; ^8^Department of Psychiatry, China Medical University Hsinchu Hospital, China Medical University, Hsinchu, China; ^9^Department of Health Services Administration, China Medical University, Hsinchu, China; ^10^Institute of Epidemiology and Preventive Medicine, College of Public Health, National Taiwan University, Taipei, Taiwan; ^11^Research Center of Big Data and Meta-Analysis, Wan Fang Hospital, Taipei Medical University, Taipei, Taiwan; ^12^Department of Anesthesiology, Chi Mei Medical Center, Tainan, Taiwan

**Keywords:** probiotics, cognitive functions, meta-analysis, neuroinflammation, child

## Abstract

**Background:**

Therapeutic efficacies of probiotics in improving neurocognitive functions in infants and young children remained unclear. This meta-analysis focused on different cognitive outcomes in this population.

**Methods:**

Major databases were searched electronically from inception to October 2023 to identify randomized controlled trials (RCTs) that investigated the therapeutic efficacy of probiotics in enhancing cognitive functions assessed by standardized tasks. The overall effect size was calculated as standardized mean difference (SMD) based on a random effects model.

**Results:**

Nine RCTs with 3,026 participants were identified. Both our primary and secondary results demonstrated no significant difference in neurocognitive outcomes between infants/children treated with probiotics and those receiving placebos. However, our subgroup analysis of studies that offered a probiotics treatment course of over six months demonstrated a significantly better neurocognitive outcome than placebos (SMD = 0.21, *p* = 0.03, two studies with 451 participants), but this finding was based on only two RCTs.

**Conclusion:**

Despite lack of significant therapeutic effects of probiotics on neurocognitive outcomes, our finding of a positive impact of probiotics on neurocognitive development in those undergoing treatment for over six months may provide an important direction for further investigations into the enhancement of therapeutic effects of probiotics on neurocognitive development in infants and young children.

**Systematic review registration:**

PROSPERO CRD42023463412.

## Introduction

There have been increasing investigations into the use of probiotics for treating a variety of neurodevelopmental disorders (1) and also for improvement of neurocognitive outcomes in preterm infants or children (2–7). The rationale of the therapeutic use of probiotics lies in the bidirectional communication between the gut and the central nervous system (CNS), so called gut-brain axis (8). There are a variety of neuroendocrinological pathways involved in the gut-brain axis (8), including the suppression of systemic and CNS inflammation (9, 10) as well as the modulation of many important neurotransmitters such as those in the dopamine system (11, 12). Not only is neuroinflammation known to be associated with different psychiatric disorders (13), but CNS inflammation may also play different roles in specific neurodevelopmental periods (14, 15). Therefore, the anti-inflammatory effects of some probiotics (9, 10) may offer additional benefits to those in early neurodevelopmental stages, especially for infants or young children.

Several studies have used probiotics in preterm infants to investigate their effects not only on growth and physical health but also on neurocognitive outcomes (2–6), mainly targeting neurocognitive development. Some studies also investigated the effects of probiotics on other neurocognitive functions such as attention, memory and processing speed (7, 16, 17). However, the results of those studies were inconsistent (2–7, 16–18), while some showed better neurocognitive outcomes (7, 16), most reported no significant differences in neurocognitive development between probiotic and placebo groups (2–5). Some methodological problems (e.g., high dropout rates due to prolonged follow up) (2–4), and differences in treatment strategies (e.g., duration of treatment and strains of probiotics) in many of those studies may contribute to the observed discrepancy in their findings.

Several factors such as timing of supplementation, duration of treatment, and the number of probiotics strains may influence the therapeutic effects of probiotics (19). A previous meta-analysis reported that only multiple-strain probiotics given as an early supplement (i.e., within seven days of birth) were associated with a lower risk of hearing impairment in preterm infants than placebo (19). The study also analyzed possible effects of probiotics on neurocognitive performances in preterm infants but did not find significant benefit of probiotic supplementation on neurodevelopmental outcomes (19). Nevertheless, that study only included four randomized controlled trials (RCTs) that investigated the neurocognitive outcomes in preterm infants without conducting any subgroup analysis or meta-regression to identify potential factors that may affect their therapeutic efficacies.

Therefore, the aim of the current meta-analysis is to provide updated evidence of the therapeutic effects of probiotics on enhancing different neurocognitive functions in infants and children as well as to identify important factors that may influence their treatment efficacies.

## Methods

### Protocol and registration

We conducted this meta-analysis in accordance with the Preferred Reporting Items for Systematic Reviews and Meta-Analyses (PRISMA) guidelines (20) and registered the protocol of current study in the international prospective register of systematic reviews (PROSPERO CRD42023463412).

### Search strategy and selection criteria

We searched eligible studies that investigated the effects of probiotics on neurocognitive development or performance in children in electronic databases including the PubMed, Embase, Cochrane CENTRAL, and ScienceDirect from inception to October 6, 2023. eTable 1 in Supplementary material provided detailed information about the search strategies and keywords used in each database. We did not set any restriction on language or countries. Relevant studies were also identified from reference lists of important review or literature to extend the scope of our search. The PICO (i.e., population, intervention, comparator, and outcomes) criteria of eligible study were: (1) Population: RCTs of participants aged less than 12 years, (2) Intervention: probiotics or products including probiotics used as monotherapy or supplementation, (3) Comparator: placebo, and (4) Outcome: performances on cognitive tasks including cognitive development, intellectual function, attention, inhibition and processing speed. Exclusion criteria were (1) studies that did not use interventions related to probiotics; (2) RCTs that included participants other than children or adolescents, and (3) those without outcome assessment for cognitive performances.

### Data extraction and quality assessment

Two independent authors (FL Lin and CM, Chen), who screened the titles and abstracts of identified literature by using predetermined keywords and search strategies (eTable 1 in Supplementary material), were also responsible for independent extraction of information including data on characteristics and outcomes of selected studies. Any disagreements about the eligibility or data of the included studies were resolved through discussion between the two authors. Inter-rater reliability was assessed by using Kappa coefficient (21). Any missing data were sought by sending electronic mails to contact the corresponding authors for original information. The quality of the eligible studies was rated according to Cochrane’s “risk of bias” assessment tool (22), while the level of evidence for each outcome was assessed by using the Grading of Recommendations Assessment, Development, and Evaluation (GRADE) (23). Any disagreements regarding the risk of bias and certainty of evidence ratings between the two authors were settled through discussion.

### Data synthesis and analysis

The primary outcome of the current study was intellectual and cognitive development measured by standardized assessment tools such as Bayley Scales of Infant and Toddler Development, Wechsler Preschool and Primary Scale of Intelligence (WIPPSI), and Wechsler Intelligence Scale for Children (WISC). The secondary outcomes encompassed available measurements on different domains of cognitive functions including attentional performance, inhibition, mental flexibility, processing speed and memory. We used Review Manager 5 (RevMan 5.4; Copenhagen: The Nordic Cochrane Center, The Cochrane Collaboration, 2014) for data analysis. As different assessment tools may be used for the same domain of cognitive functions, we chose a random effects model and standardized mean difference (SMD) to give an overall estimation of the effect size (ES) for representing the therapeutic effects of probiotics for outcome measurements of continuous data. Regarding the choice of assessment tool using RevMan 5.4, we adopted the default setting, Cohen’s d, to measure the ES, which was considered to be low, moderate, and large for values of less than 0.2, 0.5, and 0.8, respectively. To further assess the potential effects of different assessment tools on our study outcomes, we also conducted sensitivity analyses by pooling effect sizes of our included studies that used the same assessment tools. We further conducted subgroup analyses focusing on different durations of probiotics treatment and numbers of probiotic strains used (i.e., single vs. multiple strain probiotics) to investigate possible factors that may influence the therapeutic outcomes. A leave-one-out sensitivity analysis was used to assess the reliability and robustness of outcomes, while an *I*-squared test was used for heterogeneity assessment across the included studies. Statistical significance was set at a *p* value less than 0.05 for all outcome data. Finally, publication bias was assessed by inspection of a funnel plot.

## Results

### Study selection and characteristics of included studies

**Figure 1:**
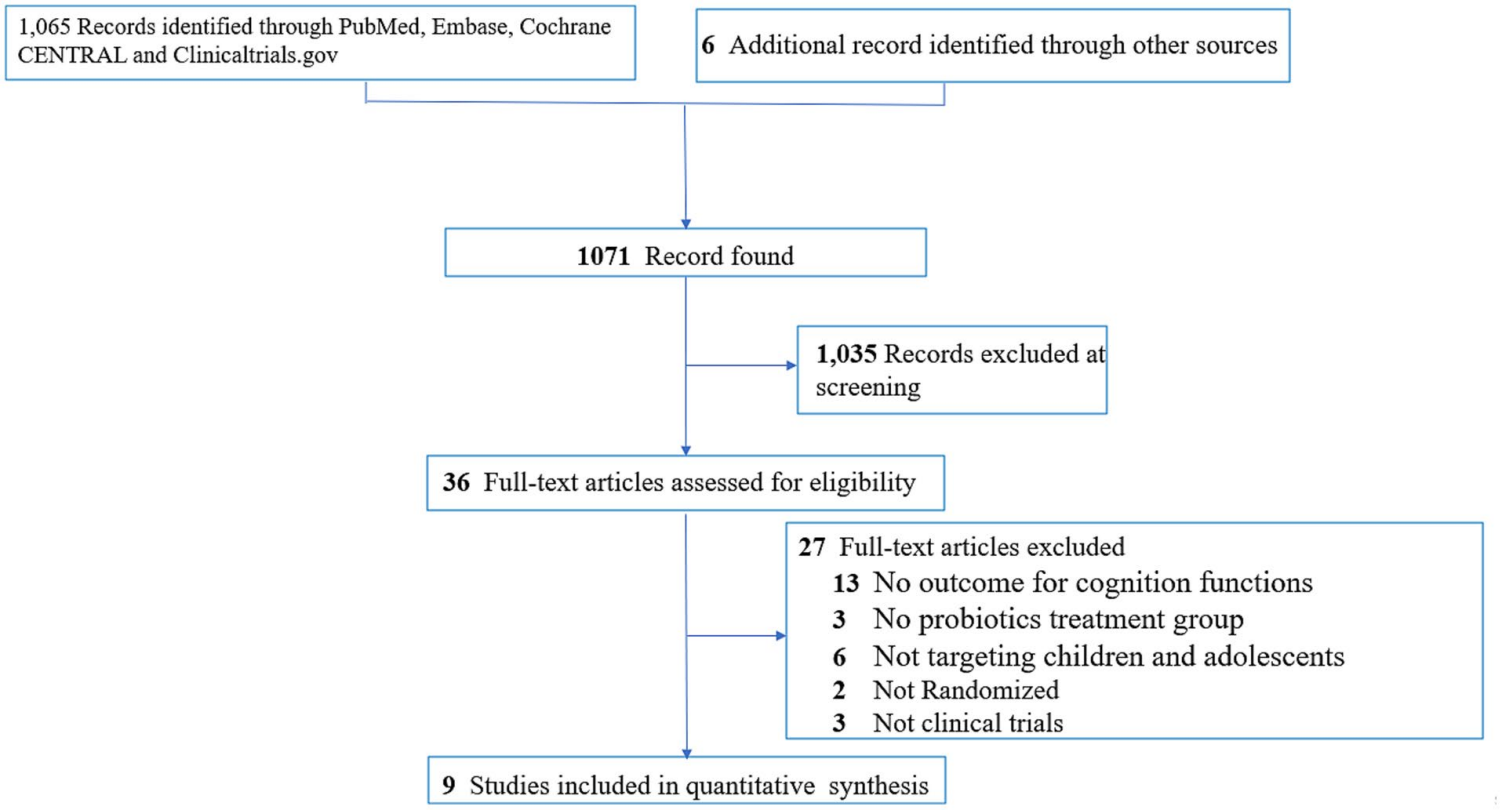
PRISMA diagram of identifying eligible studies. ADHD, attention deficit/hyperactivity disorder.

The process of study selection was conducted according to the PRISMA statement (20) (Figure 1). In brief, of the 1,071 articles initially identified from the electronic databases and relevant reviews (eTable 2 in Supplementary material), 36 were selected for full-text review after exclusion of 1,035 studies from screening of titles and abstracts. Finally, nine studies with 3,026 participants were deemed eligible according to our inclusion criteria (2–7, 16–18). The kappa coefficient for study eligibility was 1. Relevant data from the selected studies were extracted on October 6, 2023.

A summary of the characteristics of the included studies is provided in Table 1. Five out of the nine studies recruited preterm infants (2–6), one recruited participants with the diagnosis of Tourette syndrome (7) and three enrolled participants from the general population (16–18). With regard to the age of receiving probiotics, six out of the nine studies started their intervention around the time of birth (2–6, 17), while the remaining three studies started intervention at the age of one (18), five (16), and 10 years (7). Although the duration of treatment varied, most studies had a duration of treatment less than six months except two (> 6 months) (17, 18). Three out of the nine studies conducted their assessments immediately after interventions (7, 16, 18), whereas six scheduled follow-up assessment within various post-treatment time frames (i.e., six months after cessation of probiotics treatment) (2–6, 17). Four out of the nine studies adopted single strain probiotics (2, 4, 5, 7) while five used multiple strain probiotics (3, 6, 16–18). The geographic locations of the eligible trials included mainly Southeast Asia (6, 7, 18), Australia and New Zealand (2, 3, 17), and Middle Eas (4, 5) with one being conducted in Africa (16). Detailed information about different assessment tools used in each included study is provided in Table 1 and eTable 3 in Supplementary material.

**Table 1:** Summary of characteristics of studies in the current meta-analysis.

Study (year)	Diagnosis (criteria)	Design	Comparison	N	Duration (weeks)	Outcome	Time of assessment	Mean age starting treatment (years)	Female (%)	Country
Brett et al. (16)	School children	RCT	Probiotics: *Lactobacillus rhamnosus* yoba 2-9E7 cfu/mL and *Streptococcus thermophilus* 0.8-2E9 cfu/mL	87	20	Attention – Go/no go Inhibition – flanker test Flexibility – Set shifting Processing speed – Cancellation task	Immediate after intervention	5.51 (4–7)	55.38	Côte d’Ivoire
			Placebo (containing *Streptococcus thermophilus* 0.8-2E9 cfu/mL)	82						
Wu et al. (7)	Tourette syndrome (DSM-5)	RCT	Probiotics: *Lactobacillus plantarum* 9×10^9^ cfu/day	28	8	Attention – CPT Inhibition – CPT Flexibility – CPT Processing speed – CPT	Immediate after intervention	9.86 (5–18)	15.79	Taiwan
			Placebo	29						
Agrawal et al. (2)	Preterm neonates (born<33 weeks)	RCT	Probiotics: *Bifidobacterium breve* 3 × 10^9^ cfu/day	36	12 days	Cognition – MSEL Attention – NEPSY-II Inhibition – NEPSY-II Processing speed – NEPSY-II	Follow up at 3–5 years	N/A	43.3	Australia
			Placebo	31						
Slykerman et al. (17)	Infant	RCT	Probiotics: *Lactobacillus rhamnosus* 6×10^9^ cfu/day or *Bifidobacterium animalis* subsp. Lactis 9×10^9^ cfu/day	201	104	Cognition – WISC-IV Attention – CPT Inhibition – CPT Flexibility – CPT Processing speed – CPT	Follow up at 11 years	Treatment started at 35 gestation	N/A	New Zealand
			Placebo	97						
Jacobs et al. (3)	very low birth weight preterm infants (<32 completed weeks’ gestation and weighing <1,500 g)	RCT	Probiotics: *Bifidobacterium infantis* 3×10^9^ cfu, *Streptococcus thermophilus* 3.5×10^9^ cfu and *Bifidobacterium lactis* 3.5×10^9^ cfu with 1 × 109 total organisms per 1.5 g in a maltodextrin base powder.	548	Until discharge	Cognition – Bayley scale of Infant Development III	Follow up 2 years later	2 days	45.6	Australia and New Zealand
			Placebo	551						
Akar et al. (4)	very low birth weight preterm infants (gestational age ≤ 32 weeks or birth weight ≤ 1,500 g)	RCT	Probiotics: *Lactobacillus reuteri* 1×10^9^ organism/day	200	4.1	Cognition – Bayley scale of Infant Development II	Follow up at 18 to 24 month	1 day	46.6	Turkey
			Placebo	200						
Sari et al. (5)	very low birth weight preterm infants (a gestational age < 33 weeks or birth weight < 1,500 g)	RCT	Probiotics: Lactobacillus sporogenes 3.5×10^9^ cfu/day	121	5.1	Cognition – Bayley scale of Infant Development II	Follow up at 18 to 22 months	1.7 day	45.4	Turkey
			Placebo	121						
Firmansyah et al. (18)	Healthy	RCT	Synbiotics: ifidobacterium longum 1×10^7^ cfu/100 g and Lactobacillus rhamonosus 2×10^7^ cfu/100 g	199	52	Cognition – Bayley scale of Infant Development III	Immediate after intervention	1.03	48.4	Indonesia
			Placebo	194						
Chou et al. (6)	very low birth weight preterm infants (a gestational age < 33 weeks or birth weight < 1,500 g)	RCT	Probiotics: *Lactobacillus acidophilus* 2×10^9^ cfu/day and 2×10^9^ cfu/day and Bifidobacteria infantis	153	6.7	Cognition – Bayley scale of Infant Development II	Follow up at 3 years	After 7 days of birth	44.2	Taiwan
			Placebo	148						

CPT, continuous performance task; cfu, colony-forming unit; DSM-5, diagnostic and statistical manual of mental disorders fifth edition; MSEL, Mullen scales of early learning; N, number; NEPSY-II, the NEPSY second edition; RCT, randomized controlled trial; WISC-IV, Wechsler intelligence scale for children forth edition.

### Risk of bias assessment

**Figure 2:**
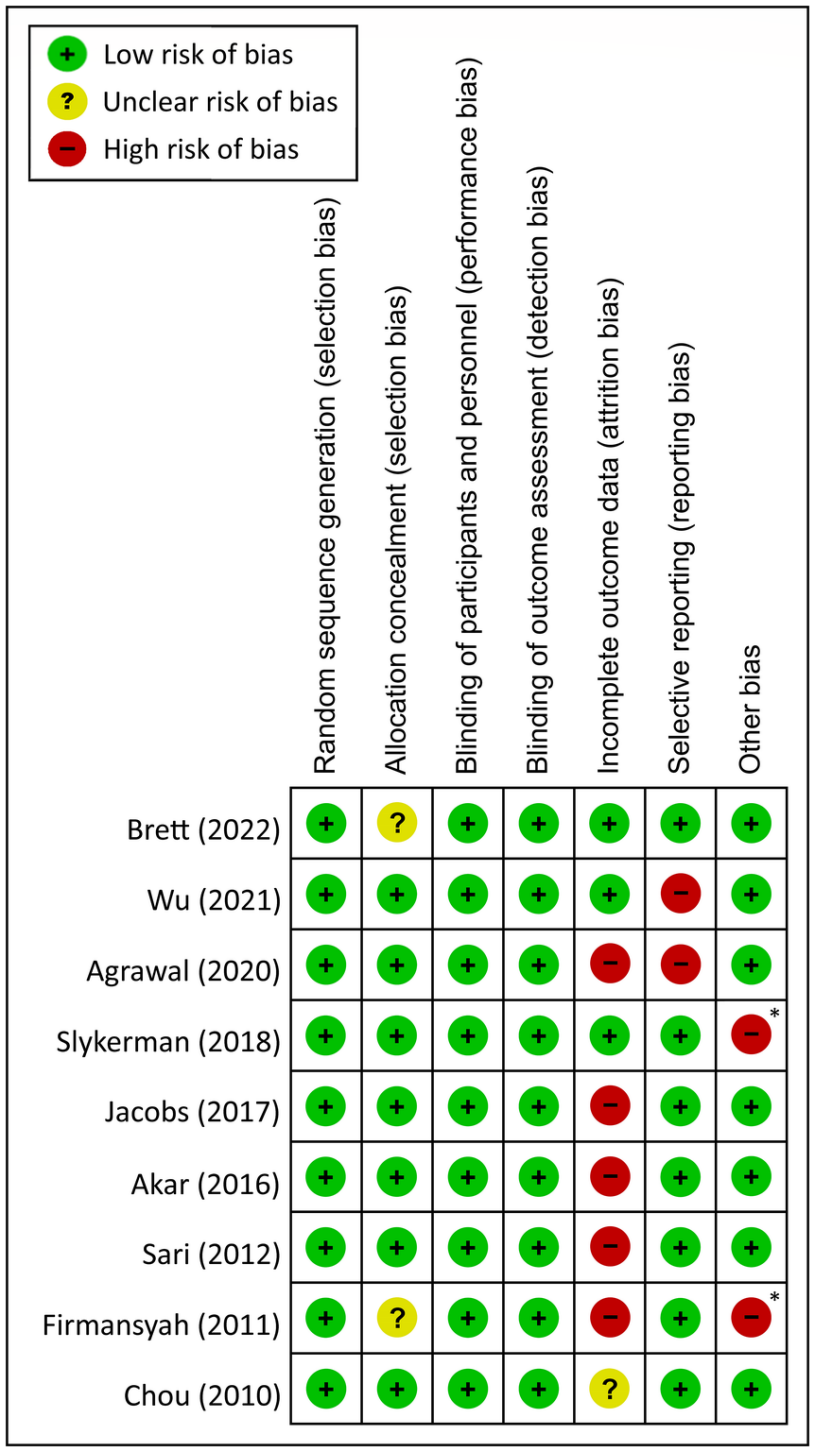
Risk of bias for eligible studies. ^X^ Sponsored by pharmaceutical company.

By using the Cochrane Collaboration’s tool for risk of bias assessment, most studies had a low or unknown risk of bias in randomization sequence and allocation concealment. Detection and performance biases were also low in most trials due to their double-blind design. However, attrition bias was found in five out of the nine studies probably due to a prolonged follow-up period (Figure 2). Two studies were considered to have a high risk of reporting bias because their primary outcomes were behavioral problems rather than cognitive functions (Figure 2). Finally, two studies were deemed at high risk of other bias because of sponsorship by private companies (17, 18) (Figure 2).

### Results of syntheses

#### Primary outcome

**Figure 3:**
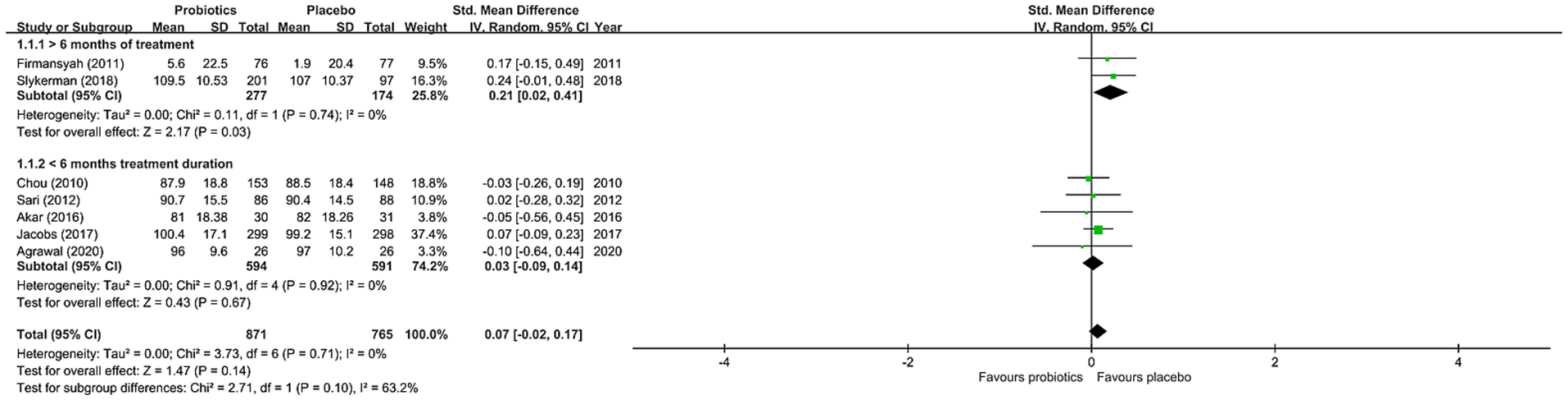
Forest plot of effect size for comparing the difference in the cognition between probiotics and placebo groups with subgroup of those receiving intervention for >6 months and that of those receiving intervention for <6 months. CI, confidence interval; Std, standardized; SE, standard error.

**Figure 4:**
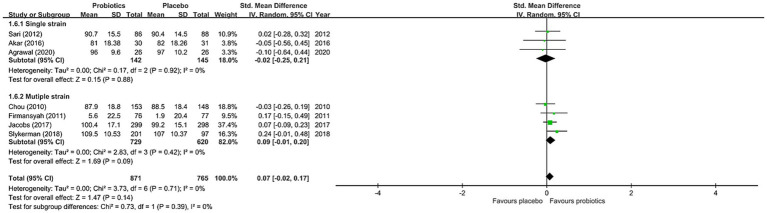
Forest plot of effect size for comparing the difference in the cognition between probiotics and placebo groups with subgroups of those receiving multiple-strain probiotics and that of those receiving single-strain probiotics. CI, confidence interval; Std, standardized; SE, standard error.

The results of the current meta-analysis showed no significant difference in neurocognitive development between the probiotics and placebo groups (SMD = 0.07, 95% CI: −0.02 to 0.17, *p* = 0.14, seven studies with 1,636 participants) (Figure 3). There was no significant heterogeneity (*I*^22^ = 0% and *p* = 0.71), inconsistency on leave-one-out sensitivity analysis, or notable asymmetry on funnel plot inspection for our primary outcomes (eFigure 1 in Supplementary material). However, our subgroup analysis focusing on studies that used probiotics for more than six months demonstrated significantly better neurocognitive development in the probiotics group than that in the placebo group (SMD = 0.21, 95% CI: 0.02 to 0.41, *p* = 0.03, two studies with 451 participants) (Figure 3). On the other hand, subgroup analysis of studies that used probiotics for less than six months showed no significant difference in cognitive performance between the probiotics and placebo groups (SMD = 0.03, 95% CI: −0.09 to 0.14, *p* = 0.92, five studies with 1,185 participants) (Figure 3). Nevertheless, no significant difference was noted when comparing the two subgroups of studies (*p* = 0.39) (Figure 3). With regard to the influence of the number of strains in the probiotic regimen on neurocognitive outcomes, subgroup analysis of studies that used single-strain probiotics demonstrated a small but non-significant difference between the probiotics and the placebo groups in favor of the latter (SMD = -0.02, 95% CI: −0.25 to 0.21, *p* = 0.88, 3 studies with 287 participants) (Figure 4). In contrast, subgroup analysis of studies that adopted multiple-strain probiotic regimens revealed apparently more favorable outcomes in the probiotics group than that in the placebo group despite the absence of statistical significance (SMD = 0.09, 95% CI: −0.01 to 0.20, *p* = 0.09, 4 studies with 1,349 participants) (Figure 4). Comparison of the two subgroups of studies showed no significant difference (*p* = 0.39) (Figure 4). Sensitivity analyses by pooling effect sizes of the studies that used the same assessment tools showed no significant change in our overall results (eTable 4 in Supplementary material).

#### Secondary outcomes

Our secondary outcomes showed no significant difference in sustained attention (SMD = −0.08, 95% CI: −0.27 to 0.11, *p* = 0.4, four studies with 464 participants), inhibition (SMD = 0.02, 95% CI: −0.23 to 0.28, *p* = 0.86, four studies with 464 participants), flexibility (SMD = -0.03, 95% CI: −0.23 to 0.18, *p* = 0.80, three studies with 404 participants), and processing speed (SMD = 0.10, 95% CI: −0.15 to 0.35, *p* = 0.45, four studies with 528 participants) between children receiving probiotic treatment and those in the placebo group (eFigures 2–5 in Supplementary material). There was no significant heterogeneity, inconsistency on leave-one-out sensitivity analysis, or notable asymmetry on funnel plot inspection for all secondary outcomes (eFigures 6–9 in Supplementary material).

#### Certainty of evidence

Grading of the certainty of evidence for individual outcomes of interest is provided in eTable 4 in Supplementary material. Our primary outcome was downgraded to low due to the limited number of eligible trials and the high risk of bias, mainly attributable to a high risk of attrition bias. The certainty of evidence was further downgraded to very low for all of our secondary outcomes because of even less available data for more precise analysis of the study outcomes.

## Discussion

Similar to the previous meta-analysis which included four RCTs (19), our study that included nine RCTs with 3,026 participants did not show significantly better neurocognitive outcomes in those receiving probiotic treatments than those in the placebo group. Our investigation into the effects of probiotics on other neurocognitive functions including attention, inhibition, processing speed, and flexibility also demonstrated no significant difference between the two groups. Nevertheless, although our subgroup analysis found no significant difference in neurocognitive development between the probiotic and placebo groups in studies that used probiotics for less than six months, significantly better neurocognitive development was noted in the probiotic group when focusing on studies that used probiotics for more than six months. Despite the need for further verification of this positive finding that was derived only from two studies (17, 18), the implication that the duration of probiotic treatment may influence neurocognitive outcome in this population may provide a direction for further investigations.

Several possible mechanisms may explain the potential association between the use of probiotics and an improvement in neurocognitive outcomes in infants and children (19). The most important hypothesis is the gut-brain-axis which involves a variety of neuroendocrine pathways underlying the bidirectional communication between gut microbiome and CNS. Both animal and human studies have reported modulating effects of certain intestinal microbiota on important neurotransmitters, such as norepinephrine and dopamine (11, 12). Communication between the central GABA system and gut microbiome through the vagus nerve was also demonstrated in an animal study (24). Moreover, given the reported negative impact of systemic inflammation on neurocognitive functions (14, 15), the systemic anti-inflammatory effects of certain probiotics (9, 10) may offer a neurocognitive benefit in this setting. Finally, several studies have demonstrated that treatment with probiotics in preterm infants may reduce the risk of late-onset sepsis (25), which was associated with a higher risk of poor cognitive development (26).

On the other hand, notwithstanding prior evidence suggesting possible positive effects of probiotics on neurodevelopment, both the results of our meta-analysis and those of another meta-analytical study (19) failed to show improved cognitive outcomes in infants and children treated with probiotics. Nevertheless, despite our inclusion of up to seven RCTs, our findings may not be robust enough to support the therapeutic benefits to neurocognitive development. Besides, methodological problems (e.g., high dropout rates due to prolonged follow-ups) and variations in treatment strategies (e.g., duration of treatment) may contribute to the finding, which needs to be verified through large-scale clinical trials with a meticulous methodological design taking into account the potential factors that may affect the therapeutic efficacies of probiotics in terms of neurocognitive functions.

A previous meta-analysis investigating the effects of probiotics on the risk of hearing development reported significant therapeutic advantage only under the condition of early supplementation (i.e., within seven days of birth) with multiple strain probiotics (19). In concert with this finding, most studies investigating neurodevelopment in our meta-analysis started probiotics within seven days of birth, except one that initiated treatment at the age of 12 months (18). Despite our inability to conduct subgroup analysis focusing on the timing of probiotics administration, our leave-one-out sensitivity test did not show a significant impact of that study (18) on the overall result. Therefore, the finding suggested that the timing of supplementation may not be a significant contributor to the efficacy of probiotics in this setting.

On the other hand, despite the lack of significant difference on subgroup analysis comparing the efficacy between studies using single-strain probiotics and those adopting multiple-strain probiotics, subgroup analysis of studies using multiple-strain probiotics showed more favorable outcomes than placebos (ES: 0.09) while studies using single-strain probiotics exhibited less favorable outcomes compared to the placebo group (ES: −0.02). Given prior evidence showing additional benefits of multiple-strain probiotics through increasing the chance of adhesion of desirable microbiota to the intestinal mucosa (27), future research is warranted to investigate the potential differences in therapeutic effects on neurocognitive development between single-strain and multiple-strain regimens.

The most intriguing finding of our meta-analysis is that our subgroup analysis of studies that offered a probiotics treatment course of more than six months demonstrated a significantly better neurocognitive outcome than placebos. Coupled with previous evidence supporting the lack of a long-lasting effect of probiotics on colonization of the gut after cessation of supplementation for 1–4 weeks (28), our findings implied that a short duration of probiotic supplementation may not be enough to exert their effects on long-term neurodevelopment. Although this result was based on only two RCTs (17, 18), its clinical implication may be substantial. Finally, the results of our secondary analysis investigating the effects of probiotics on other neurocognitive functions including attention, inhibition, processing speed, and flexibility all failed to show significant improvements in the probiotics group compared to the placebo group. Nevertheless, the GRADE of evidence was very low due to the limited numbers of eligible studies for our secondary outcomes. Further large-scale studies are required to verify these findings.

The current study had several limitations. First, because the number of RCTs focusing on the potential beneficial effects of probiotics on neurocognitive functions in children/adolescents was limited, we could only identify nine in the present meta-analysis. Of the nine studies, merely seven were available for analyzing our primary outcome with evidence supporting the positive neurocognitive outcome in the probiotics group with a treatment course over six months derived from two RCTs. Therefore, the quality of evidence was rated as low or very low in most of our findings. Further studies are required to support our results. Nevertheless, the results of our subgroup analysis on the duration of probiotics use may provide an important direction for further studies to enhance the therapeutic effects of probiotics on neurocognitive development. Second, a high risk of attrition bias due to high dropout rates in five out of the nine studies probably attributable to a prolonged follow-up period (i.e., three years) may further compromise the quality of evidence derived from the current investigation. Future studies focusing on the association between different follow-up periods and the effects of probiotics on neurocognitive functions are warranted. Third, in addition to dietary factor-related alteration in gut microbiota (29, 30), that has been reported to affect neuropsychological development due to the brain-gut axis (31), several other nutritional and metabolic factors such as vitamin deficiency (32), iron deficiency (33), glucose metabolism (34), and thyroid function (34) are also known to be associated with neuropsychiatric disorders. However, a lack of information about many of these potential confounding factors in most of the included studies precluded our conduction of further meta-regression or subgroup analysis to evaluate their influences. The inclusion of such information is recommended in future clinical trials. Fourth, variations in neuropsychological tests used in our included studies may contribute to potential heterogeneity for which we used a random effects model to minimize its influence as well as adopted sensitivity analysis by pooling effect sizes of the studies that used the same tests to evaluate its impact. Although sensitivity analysis demonstrated no notable change in the overall results, the possibility that different assessment tools may affect our study outcomes still cannot be ruled out. Fifth, although dosage and characteristics of probiotics may influence our study outcomes, relevant subgroup analyses could not be conducted due to the wide variation in strains as well as combination of the regimen (e.g., single vs. multiple strains) in addition to the relatively small number of included trials. Nevertheless, our subgroup analysis revealed more favorable neurocognitive outcomes associated with the use of multiple-strain probiotics than those related to single-strain regimens despite the lack of statistical significance. Further meta-analytical studies are warranted to address these issues. Overall, the quality of current evidence, which was rated from low to very low, could not rule out the potential positive influence of probiotics on neurocognitive functions in young children. Further large-scale investigations are warranted to provide more robust evidence.

## Conclusion

The current study showed no significant difference in neurocognitive outcomes between infants/children treated with probiotics and those receiving placebos. Nevertheless, current evidence is still not strong enough to rule out the beneficial effects of probiotics on neurocognitive development in this population due to the limited number of available studies and a high risk of attrition bias in most of the included trials. On the other hand, our finding of a positive impact of probiotics on neurocognitive development in those undergoing treatment for over six months may provide an important direction for further investigations into the enhancement of therapeutic effects of probiotics on neurocognitive development.

## Data availability statement

The original contributions presented in the study are included in the article/Supplementary material, further inquiries can be directed to the corresponding author.

## Author contributions

F-LL: Conceptualization, Formal analysis, Methodology, Writing – original draft, Writing – review & editing. C-MC: Conceptualization, Formal analysis, Software, Writing – original draft, Writing – review & editing. C-KS: Investigation, Supervision, Writing – original draft, Writing – review & editing. Y-SC: Data curation, Investigation, Writing – original draft, Writing – review & editing. R-FT: Data curation, Methodology, Writing – original draft, Writing – review & editing. H-JC: Conceptualization, Validation, Visualization, Writing – original draft, Writing – review & editing. M-YW: Formal analysis, Investigation, Writing – original draft, Writing – review & editing. Y-CC: Formal analysis, Methodology, Writing – original draft, Writing – review & editing. K-CH: Conceptualization, Supervision, Writing – original draft, Writing – review & editing.
