# Supplementary material for: Effects of probiotics on neurocognitive outcomes in infants and young children: a meta-analysis
Source: Front Public Health. 2023 Dec 5;11:1323511. doi: 10.3389/fpubh.2023.1323511 (PMC10728653; doi:10.3389/fpubh.2023.1323511)
Supplement: Supplementary file 1 [file Table_1.DOCX]

**eTable 1. Applied keywords and search results from each database**

| Database | Keywords | Filter | Date (yyyy/mm/dd) | | Result |
| --- | --- | --- | --- | --- | --- |
| PubMed | (probiotics or psychobiotic or probio) and (cognition or attention or memory or executive functions or learning or neuropsychological test or Bayley scales of infant development or Wechsler Intelligence Scale for Children or Wechsler Preschool and Primary Scale of Intelligence or Mullen Scale of Early Learning or development or Cambridge Neuropsychological Test Automated Battery or Flanker Task or attention network or stroop or N-back or go-no-go or stop signal or digit span or automated working memory assessment battery or continuous performance task) | 1.RCT  2.Child: birth – 18 years | | 2023/09/21 | 393 |
| Embase | (adhd OR hkd OR add OR Attention deficit hyperactivity disorder OR attention deficit OR hyperactivity OR inattentive OR impulsivity OR neurodevelopmental disorder) and (probiotics OR gut microbiota OR gut-brain axis OR bifidobacteria OR lactobacillus) | 1.RCT  2.Newbor, infant, children and adolescents | | 2023/09/21 | 163 |
| Cochrane CENTRAL | (probiotics or psychobiotic or probio) and (cognition or attention or memory or executive function or learning) | Trials | | 2023/09/21 | 460 |
| ScienceDirect | probiotics and (cognition or attention or memory or executive function) | Research articles | | 2023/09/21 | 49 |

Abbreviations: NA, not applied; RCT, randomized controlled trial

**eTable 2** Reasons for study exclusion

| **Reason** | **Number of excluded studies** | **References** | |
| --- | --- | --- | --- |
| No outcome for cognition functions  No probiotic treatment group | 13  3 | | [1-13]  [14-16] |
| Not targeting children and adolescents | 6 | | [17-22] |
| Not randomized | 2 | | [23, 24] |
| Not clinical trials | 3 | | [25-27] |

ADHD: attention deficit hyperactivity disorder

RCT: randomized controlled trials

**References**

[1] Sjödin KS, Sjödin A, Ruszczyński M, Kristensen MB, Hernell O, Szajewska H, et al. Targeting the gut-lung axis by synbiotic feeding to infants in a randomized controlled trial. BMC Biol. 2023;21(1):38. <https://doi.org/10.1186/s12915-023-01531-3>.

[2] Zhang L, Xu Y, Li H, Li B, Duan G, Zhu C. The role of probiotics in children with autism spectrum disorders: A study protocol for a randomised controlled trial. PLoS One. 2022;17(2):e0263109. <https://doi.org/10.1371/journal.pone.0263109>.

[3] Mageswary MU, Ang XY, Lee BK, Chung YF, Azhar SNA, Hamid IJA, et al. Probiotic Bifidobacterium lactis Probio-M8 treated and prevented acute RTI, reduced antibiotic use and hospital stay in hospitalized young children: a randomized, double-blind, placebo-controlled study. Eur J Nutr. 2022;61(3):1679-91. <https://doi.org/10.1007/s00394-021-02689-8>.

[4] Kong XJ, Liu J, Liu K, Koh M, Sherman H, Liu S, et al. Probiotic and Oxytocin Combination Therapy in Patients with Autism Spectrum Disorder: A Randomized, Double-Blinded, Placebo-Controlled Pilot Trial. Nutrients. 2021;13(5). <https://doi.org/10.3390/nu13051552>.

[5] Qin Q, Liu H, Yang Y, Wang Y, Xia C, Tian P, et al. Probiotic Supplement Preparation Relieves Test Anxiety by Regulating Intestinal Microbiota in College Students. Dis Markers. 2021;2021:5597401. <https://doi.org/10.1155/2021/5597401>.

[6] Martí M, Spreckels JE, Ranasinghe PD, Wejryd E, Marchini G, Sverremark-Ekström E, et al. Effects of Lactobacillus reuteri supplementation on the gut microbiota in extremely preterm infants in a randomized placebo-controlled trial. Cell Rep Med. 2021;2(3):100206. <https://doi.org/10.1016/j.xcrm.2021.100206>.

[7] Nieto-Ruiz A, Diéguez E, Sepúlveda-Valbuena N, Herrmann F, Cerdó T, López-Torrecillas F, et al. The Effects of an Infant Formula Enriched with Milk Fat Globule Membrane, Long-Chain Polyunsaturated Fatty Acids and Synbiotics on Child Behavior up to 2.5 Years Old: The COGNIS Study. Nutrients. 2020;12(12). <https://doi.org/10.3390/nu12123825>.

[8] Cui X, Shi Y, Gao S, Xue X, Fu J. Effects of Lactobacillus reuteri DSM 17938 in preterm infants: a double-blinded randomized controlled study. Ital J Pediatr. 2019;45(1):140. <https://doi.org/10.1186/s13052-019-0716-9>.

[9] Maldonado J, Gil-Campos M, Maldonado-Lobón JA, Benavides MR, Flores-Rojas K, Jaldo R, et al. Evaluation of the safety, tolerance and efficacy of 1-year consumption of infant formula supplemented with Lactobacillus fermentum CECT5716 Lc40 or Bifidobacterium breve CECT7263: a randomized controlled trial. BMC Pediatr. 2019;19(1):361. <https://doi.org/10.1186/s12887-019-1753-7>.

[10] Wejryd E, Marchini G, Frimmel V, Jonsson B, Abrahamsson T. Probiotics promoted head growth in extremely low birthweight infants in a double-blind placebo-controlled trial. Acta Paediatr. 2019;108(1):62-9. <https://doi.org/10.1111/apa.14497>.

[11] Santocchi E, Guiducci L, Fulceri F, Billeci L, Buzzigoli E, Apicella F, et al. Gut to brain interaction in Autism Spectrum Disorders: a randomized controlled trial on the role of probiotics on clinical, biochemical and neurophysiological parameters. BMC Psychiatry. 2016;16:183. <https://doi.org/10.1186/s12888-016-0887-5>.

[12] Nieto-Ruiz A, Cerdó T, Jordano B, Torres-Espínola FJ, Escudero-Marín M, García-Ricobaraza M, et al. Maternal weight, gut microbiota, and the association with early childhood behavior: the PREOBE follow-up study. Child and Adolescent Psychiatry and Mental Health. 2023;17(1). <https://doi.org/10.1186/s13034-023-00589-9>.

[13] Patole S, Keil AD, Chang A, Nathan E, Doherty D, Simmer K, et al. Effect of Bifidobacterium breve M-16V supplementation on fecal bifidobacteria in preterm neonates--a randomised double blind placebo controlled trial. PLoS One. 2014;9(3):e89511. <https://doi.org/10.1371/journal.pone.0089511>.

[14] Wang J, Liu Y, Huang HY, Wu JT, Wang WJ. [Influence of acupuncture on the clinical manifestations and gastrointestinal symptoms of children with autism spectrum disorder]. Zhongguo zhen jiu = Chinese acupuncture & moxibustion. 2022;42(12):1373-6. <https://doi.org/10.13703/j.0255-2930.20220111-0004>.

[15] Singh I, Behera DP, T KA, Gupta S. Efficacy and safety of tamsulosin vs its combination with mirabegron in the management of lower urinary tract non-neurogenic overactive bladder symptoms (OABS) because of Benign Prostatic Enlargement (BPE)-An open label randomised controlled clinical study. Int J Clin Pract. 2021;75(7):e14184. <https://doi.org/10.1111/ijcp.14184>.

[16] Smits MM, Fluitman KS, Herrema H, Davids M, Kramer MHH, Groen AK, et al. Liraglutide and sitagliptin have no effect on intestinal microbiota composition: A 12-week randomized placebo-controlled trial in adults with type 2 diabetes. Diabetes Metab. 2021;47(5):101223. <https://doi.org/10.1016/j.diabet.2021.101223>.

[17] Zhang X, Yang H, Zhang K, Zhang J, Lu X, Guo H, et al. Effects of exercise or tai chi on Internet addiction in college students and the potential role of gut microbiota: A randomized controlled trial. Journal of affective disorders. 2023;327:404-15. <https://doi.org/10.1016/j.jad.2023.02.002>.

[18] Schaub AC, Schneider E, Vazquez-Castellanos JF, Schweinfurth N, Kettelhack C, Doll JPK, et al. Clinical, gut microbial and neural effects of a probiotic add-on therapy in depressed patients: a randomized controlled trial. Transl Psychiatry. 2022;12(1):227. <https://doi.org/10.1038/s41398-022-01977-z>.

[19] Carlos LO, Ramos MRZ, Wagner NRF, Freitas LAC, Felicidade I, Campos ACL. PROBIOTIC SUPPLEMENTATION ATTENUATES BINGE EATING AND FOOD ADDICTION 1 YEAR AFTER ROUX-EN-Y GASTRIC BYPASS: A RANDOMIZED, DOUBLE-BLIND, PLACEBO-CONTROLLED TRIAL. Arquivos brasileiros de cirurgia digestiva : ABCD = Brazilian archives of digestive surgery. 2022;35:e1659. <https://doi.org/10.1590/0102-672020210002e1659>.

[20] Cardona D, Roman P, Cañadas F, Sánchez-Labraca N. The Effect of Multiprobiotics on Memory and Attention in Fibromyalgia: A Pilot Randomized Controlled Trial. Int J Environ Res Public Health. 2021;18(7). <https://doi.org/10.3390/ijerph18073543>.

[21] OjiNjideka Hemphill N, Pezley L, Steffen A, Elam G, Kominiarek MA, Odoms-Young A, et al. Feasibility Study of Lactobacillus Plantarum 299v Probiotic Supplementation in an Urban Academic Facility among Diverse Pregnant Individuals. Nutrients. 2023;15(4). <https://doi.org/10.3390/nu15040875>.

[22] Severance EG, Gressitt KL, Stallings CR, Katsafanas E, Schweinfurth LA, Savage CLG, et al. Probiotic normalization of Candida albicans in schizophrenia: A randomized, placebo-controlled, longitudinal pilot study. Brain Behav Immun. 2017;62:41-5. <https://doi.org/10.1016/j.bbi.2016.11.019>.

[23] Wang LJ, Yang CY, Kuo HC, Chou WJ, Tsai CS, Lee SY. Effect of Bifidobacterium bifidum on Clinical Characteristics and Gut Microbiota in Attention-Deficit/Hyperactivity Disorder. J Pers Med. 2022;12(2). <https://doi.org/10.3390/jpm12020227>.

[24] Wallace CJK, Milev RV. The Efficacy, Safety, and Tolerability of Probiotics on Depression: Clinical Results From an Open-Label Pilot Study. Frontiers in Psychiatry. 2021;12.

[25] Wang L, Cai Y, Garssen J, Henricks PAJ, Folkerts G, Braber S. The Bidirectional Gut-Lung Axis in COPD. Am J Respir Crit Care Med. 2023. <https://doi.org/10.1164/rccm.202206-1066TR>.

[26] Rianda D, Agustina R, Setiawan EA, Manikam NRM. Effect of probiotic supplementation on cognitive function in children and adolescents: a systematic review of randomised trials. Beneficial microbes. 2019;10(8):873-82. <https://doi.org/10.3920/bm2019.0068>.

[27] Kalenik A, Kardaś K, Rahnama A, Sirojć K, Wolańczyk T. Gut microbiota and probiotic therapy in ADHD: A review of current knowledge. Prog Neuropsychopharmacol Biol Psychiatry. 2021;110:110277. <https://doi.org/10.1016/j.pnpbp.2021.110277>.

**eTable 3. Detailed information on each assessment tool included in this meta-analysis for outcome measurements**

| **Assessment tools** | **Content** | **Reference** |
| --- | --- | --- |
| Bayley scale of Infant Development | The Bayley Scales of Infant and Toddler Development is an instrument for assessing cognitive, language, and motor functions as well as social-emotional and adaptive behavior development in infants and young children. | 1 |
| MSEL | An assessment battery for measuring development in infants and preschool children with age ranging from birth to 68 months. | 2 |
| WISC-IV | A standardized tool for assessing children’s intellectual ability. | 3 |
| CPT | A computer-administered test for evaluating problems with inhibition, flexibility, attention, and processing speed. | 4 |
| NEPSY-II | A standardized neuropsychological battery for assessing functioning in six domains: memory and learning, executive functioning and attention, social perception, sensorimotor functioning, language, and visuospatial processing in children aged 3–16 years. | 5 |
| Go/no go | A component of Continuous Performance Task, in which children are required to pressed a button on seeing a target image shown in a series of distracting images to measure their sustained and selective attention as well as response inhibition. | 6 |
| flanker test | A measure of response inhibition that involves showing children a horizontal line of five fish and instructing them to press the arrow key to indicate the direction in which the middle fish was facing that could be congruent or incongruent with that of the other fish. | 7 |
| Set shifting | To measure cognitive flexibility, children are instructed to match a colored shape (e.g., blue triangle) in the middle of the screen to one of two colored shapes (e.g., red triangle and a blue rectangle) at the bottom of the screen with either a shape rule or a color rule. | 8 |
| Cancellation task | A cancellation task to measure selective attention and processing speed in which children are given 60 seconds to delete as many target items (e.g., apples) as possible (a total of 35) out of a field with approximately 250 distractor items (e.g., other types of fruit). | 9 |

CPT: continuous performance task; MSEL: Mullen Scales of Early Learning; N: number; NEPSY-II: The NEPSY, Second Edition; WISC- IV: Wechsler Intelligence Scale for Children, Fourth Edition

**References**

1. Bayley N. Bayley Scales of Infant and Toddler Development, The Psycholgical Corporation. 3rd ed. Harcourt Assessment, Inc, 2006.
2. E.M. Mullen, Mullen Scales of Early Learning: AGS Circle Pines, MN, (1995).
3. Wechsler D. Wechsler Intelligence Scale for Children Fourth Edition. San Antonio, TX: Psychological Corporation. 2003.
4. Conners, C.K.; Sitarenios, G. Conners’ Continuous Performance Test (CPT). In Encyclopedia of Clinical Neuropsychology; Kreutzer, J.S., DeLuca, J., Caplan, B., Eds.; Springer: New York, NY, USA, 2011; pp. 681–683.
5. M. Korkman, V. Kirk, S. Kemp, Clinical and Interpretive Manual, PsychCorp, San Antonio, TX, 2007.
6. Kirmizi-Alsan, E. et al. Comparative analysis of event-related potentials during Go/NoGo and CPT: Decomposition of electrophysiological markers of response inhibition and sustained attention. Brain Res. 1104(1), 114–128 (2006).
7. Eriksen, B. A. & Eriksen, C. W. Effects of noise letters upon the identification of a target letter in a nonsearch task. Percept. Psychophys. 16(1), 143–149 (1974).
8. Hooper, S. R., Swartz, C. W., Wakely, M. B., de Kruif, R. E. L. & Montgomery, J. W. Executive functions in elementary school children with and without problems in written expression. J. Learn. Disabil. 35(1), 57–68 (2002).
9. Wechsler, D. Wechsler Intelligence Scale for Children, 5th ed. (PsychCorp, 2014).

**eTable 4. Sensitivity analyses of studies using same assessment tools**

| Outcomes | Number of studies | Effect sizes (95%CI) | Effect size p value | Heterogeneity I^2^ (%) |
| --- | --- | --- | --- | --- |
| Cognition (Bayley scale of Infant Development) | 5 | 0.05 (-0.06; 0.16) | 0.4 | 0% |
| Sustained attention (Continuous Performance Test) | 2 | -0.03 (-0.26; 0.19) | 0.76 | 0% |
| Inhibition (Continuous Performance Test) | 2 | 0.18 (-0.04; 0.40) | 0.12 | 63.4% |
| Flexibility (Continuous Performance Test) | 2 | -0.01 (-0.24; 0.21) | 0.9 | 0% |
| Processing speed (Continuous Performance Test) | 2 | 0.11 (-0.11; 0.33) | 0.33 | 63.4% |

**eTable 5.** Grading of Recommendations Assessments, Development and Evaluation (GRADE) assessment of the strength of evidence for standard weighted meta-analysis

| Outcome | Design | Risk of bias | Indirectness | Inconsistency | Imprecision | Publication bias | Grade Quality |
| --- | --- | --- | --- | --- | --- | --- | --- |
| Cognition | RCT x 7 | Serious | No indirectness | No serious  inconsistency | Serious  imprecision | Undetected | ⊕⊕ΟΟ^2,4^ |
| Attention | RCT x 4 | Serious | No indirectness | No serious  inconsistency | Serious  imprecision | Undetected | ⊕ΟΟΟ^2*,4^ |
| Inhibition | RCT x 4 | Serious | No indirectness | No serious  inconsistency | Serious  imprecision | Undetected | ⊕ΟΟΟ^2*,4^ |
| Flexibility | RCT x 3 | Serious | No indirectness | No Serious  inconsistency | Serious  imprecision | Undetected | ⊕ΟΟΟ^2*,4^ |
| Processing speed | RCT x 4 | Serious | No indirectness | No Serious  inconsistency | Serious  imprecision | Undetected | ⊕ΟΟΟ^2*,4^ |

*Risk of bias was estimated using Cochrane risk of bias, studies were classified as having low risk of bias if none of the domains above was rated as high risk of bias and three or less were rated as unclear risk; moderate if one was rated as high risk of bias or none was rated as high risk of bias but four or more were rated as unclear risk, and all other cases were assumed to pertain to high risk of bias.

Down-graded due to: ^1^ risk of bias, ^2^ indirectness, ^3.^ Inconsistency, ^4.^ Imprecision, ^5.^ publication bias

^2*^ Down-graded two levels due to very low sample sizes

**GRADE Working Group grades of evidence:**
-**High certainty**: We are very confident that the true effect lies close to that of the estimate of the effect
-**Moderate certainty**: We are moderately confident in the effect estimate: The true effect is likely to be close to the estimate of the effect, but there is a possibility that it is substantially different.
-**Low certainty**: Our confidence in the effect estimate is limited: The true effect may be substantially different from the estimate of the effect.
-**Very low certainty**: We have very little confidence in the effect estimate: The true effect is likely to be substantially different from the estimate of effect.

**eFigure 1. Funnel plot – neurocognitive development**


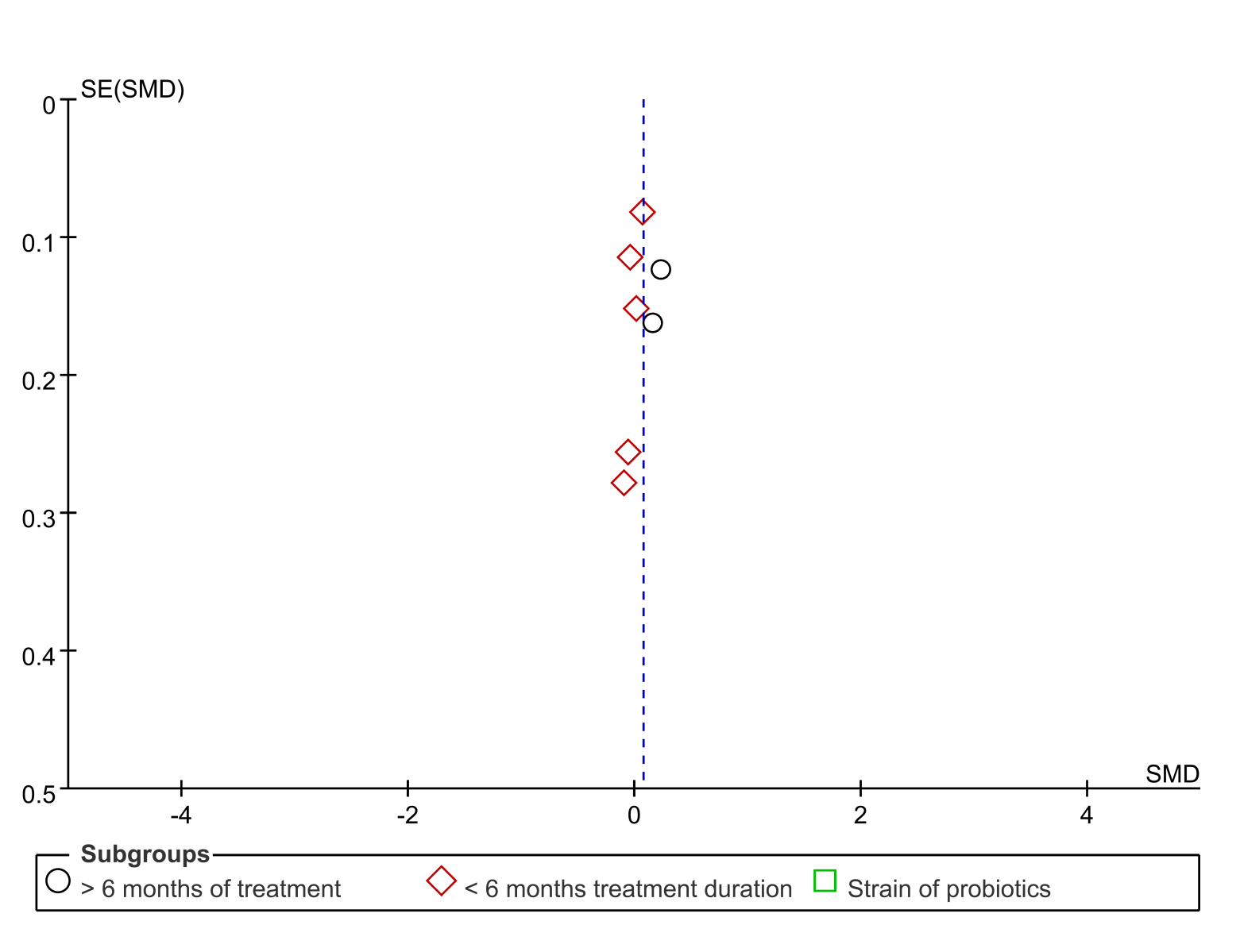


**eFigure 2. Forest plot of effect size for comparing the difference in sustained attention between probiotics and placebo groups**


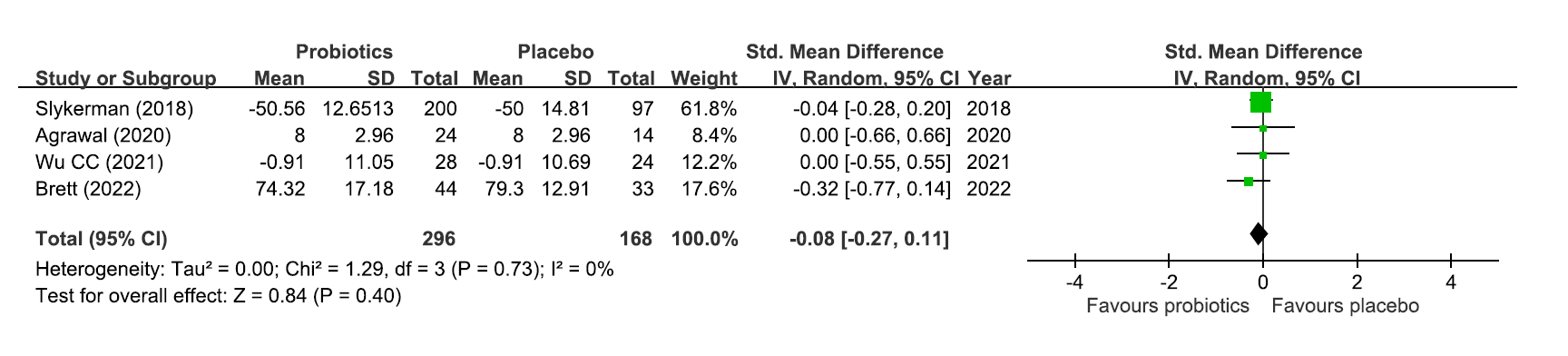


**eFigure 3. Forest plot of effect size for comparing the difference in inhibition between probiotics and placebo groups**


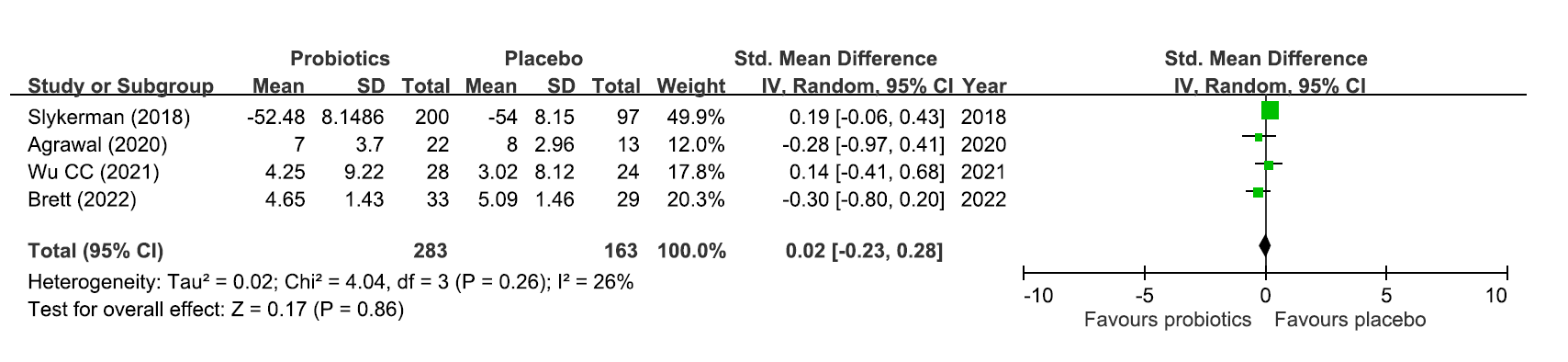


**eFigure 4. Forest plot of effect size for comparing the difference in flexibility between probiotics and placebo groups**


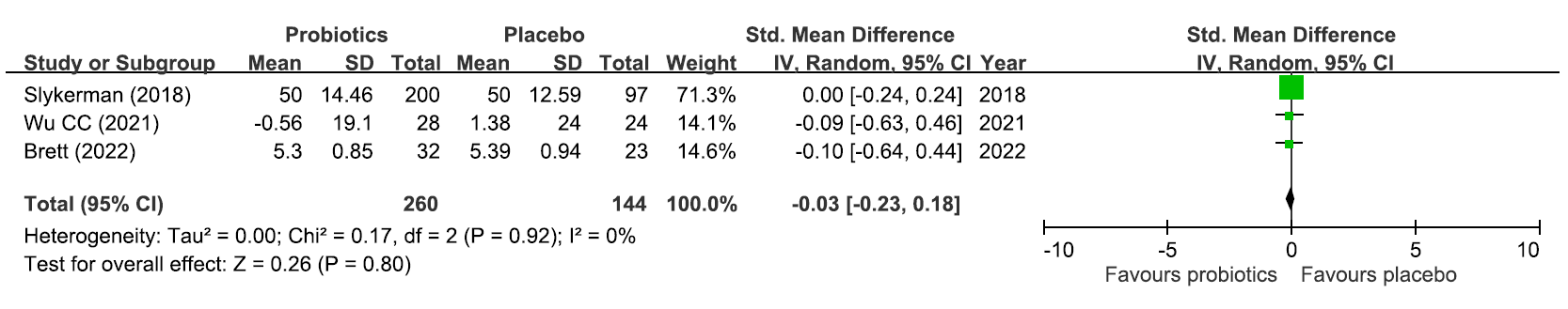


**eFigure 5. Forest plot of effect size for comparing the difference in processing speed between probiotics and placebo groups**


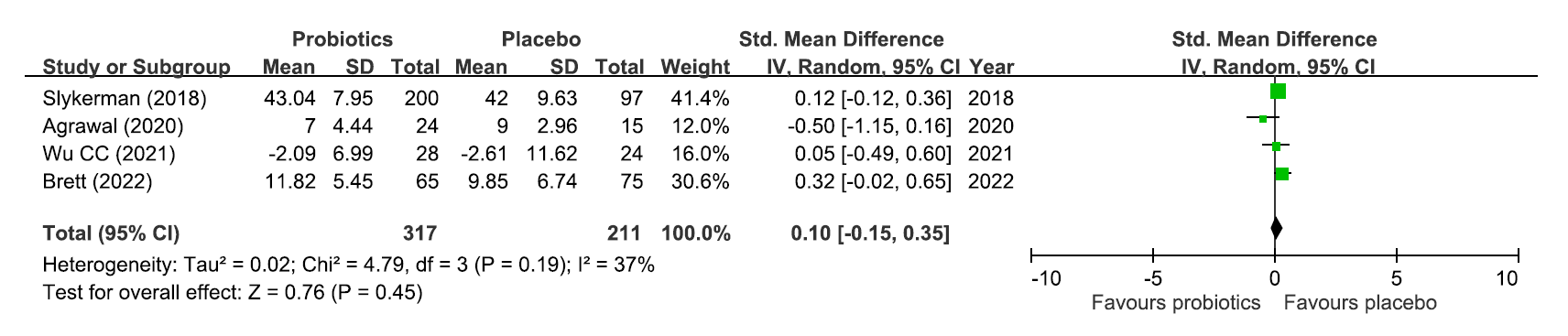


**eFigure 6. Funnel plot – sustained attention**


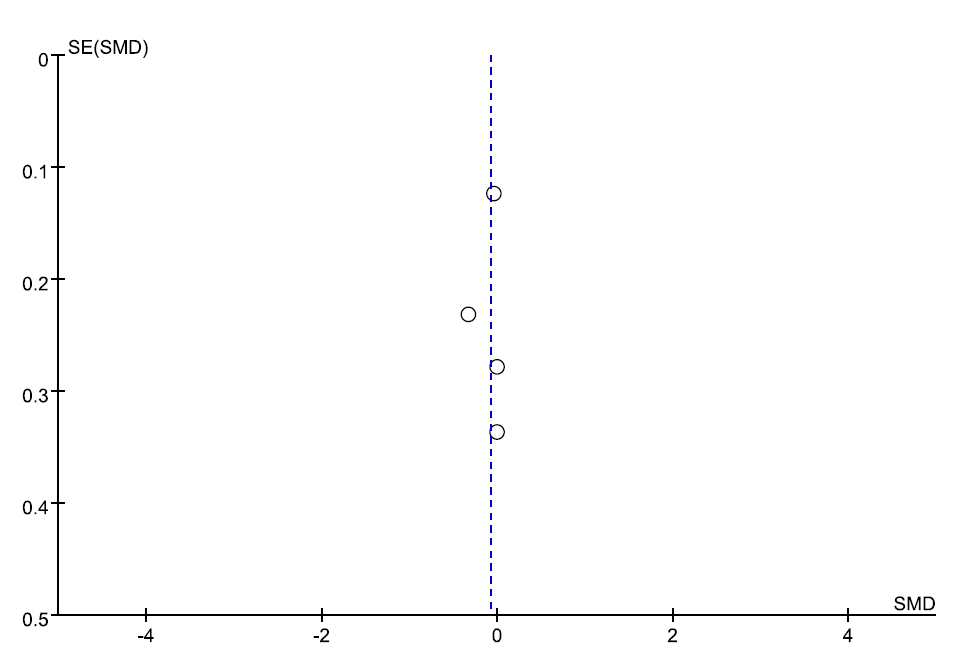


**eFigure 7. Funnel plot – inhibition**

**eFigure 8. Funnel plot – flexibility**

**eFigure 9. Funnel plot – processing speed**
